# Dentists and the Census

**Published:** 1892-06

**Authors:** 


### Editorial, Etc.

DENTISTS AND THE CENSUS.—The April number noted that a bill had been introduced into Congress in March providing penalties for refusal to answer questions included in the schedules of the Census Office, and the May number referred to the unanimous action of the Maryland State Dental Association in appointing at its ninth annual session in May a committee to convey to the Senators and Representatives of Maryland in Congress the protest of the dentists "against the action of the Census Bureau in classifying dentists as manufacturers."

This committee acted promptly in the matter, visiting

Washington on several occasions and agreed upon an amendment to the Bill to be introduced before it was reported to the Senate (as it had already passed the House of Representatives), providing that nothing in the Act "shall be construed as authority to collect statistics from professional men such as lawyers, physicians and dentists."

The House Bill, it should be stated, gave the Secretary of the Interior and his subordinates in the Census Bureau the authority to include dentists in the schedule of manufacturers, and to compel them under severe and unprecedented penalties to give detailed statements of their entire business.

The Committee was finally given a hearing before the Senate Committee on the Census. with a view to having the amendment referred to added to the bill, so that if it became a law it would not affect dentists. The matter was so effectually put not only by the representatives from Maryland. but also by committees from District of Columbia, Pennsylvania and New Jersey, that the Superintendent of the Census agreed in writing "with the representatives of the Dental Association present at a meeting of the Committee on the Census to carry out the spirit and purposes" of the amendment.

Following is a copy of the memorial presented:

*To the Senators and Representatives of Maryland and other States, in Congress Assembled:*

GENTLEMEN:—

At the last annual meeting of the Maryland State Dental Society, held in Baltimore, on May 9th and 10th, the following resolution was unanimously adopted:

"WHEREAS, a bill has been introduced in Congress, which provides that any person who shall neglect, or refuse to give information to census officers, shall be subject to a fine, "not exceeding ten thousand dollars, to which may be added imprisonment, for a period not exceeding one year," and whereas the public press reports that the bill referred to, "will be used in the nature of thumbscrews and spiked boots to enforce replies," and further, that "the dentists of Baltimore, who by their refusal to answer certain questions * *

* * * * will be the first victims of the inquisitorial machine if the bill is passed;" therefore,

RESOLVED, That this Association re-affirms the resolution unanimously passed November, 1890, protesting against the action of the Census Bureau, in classifying dentists as manufacturers and refusing to fill out blanks.

RESOLVED further, that the chairmen of the several standing committees, as now constituted, be appointed to convey the sentiments of this Association to the Senators and Representatives of Maryland in Congress, and urge them to have the bill referred to amended, so as to omit dentistry in all its branches, from the tabulations of the census."

We therefore, the undersigned, the committee appointed in accordance with this resolution, respectfully submit the following to your consideration:

1. Dentistry is recognized as a profession by law. Dentists are not required to serve on juries; they have been recognized as professional men, to wit: in the case of Noble, before Judge McArthur of Washington city. The dentists of Baltimore have always been excused from jury duty.

2. Nearly all States of the Union have passed laws recognizing them as professional men, and in some of the States license fees are demanded from both physicians and dentists for the privilege of practicing, and both are equally amenable to the law of malpractice.

3. Most of the States have appointed examining boards, to test the fitness of candidates for practice. Similar laws are made bearing on the medical profession.

4. Maryland, Pennsylvania, New York, Massachusetts, Michigan, Minnesota, Kansas, Iowa, Illinois, Indiana, Missouri, Tennessee, Georgia and Louisiana, have through their respective Legislatures granted charters to dental colleges,giving them authority to educate students in dentistry, and to confer upon those properly qualified the professional degree of Doctor of Dental Surgery.

5. In the National University's dental department in the city of Washington, D. C., the diplomas are signed by the President of the United States. This we think originated with General Grant.

6. Almost all the great Universities including those of the city of Washington, have added dental departments, and are granting professional degrees by the authority of the United States Government.

7. The American Medical Association has recognized dentists as specialists in medicine, and the International Medical Congress of the whole civilized world has done the same.

8. Dentists have given to the world the first anæsthetics, both nixtrous oxide gas and ether, which Dr. Oliver Wendell Holmes has declared "the greatest boon ever given for humanity." They have also furnished the first splint really satisfactory in the treatment of fractured jaws. This splint was applied in the case of the Hon. W. H. Seward, with entirely satisfactory results, and this after the medical profession had utterly failed to reduce the fracture.

9. Both general and oral surgery are impossible without mechanic, and all operations both in general and oral surgery are more or less mechanical.

10. In the past, both surgery and dentistry were classed among the trades; but time, progress, cultivation and advancement have altered the condition of things, and to-day surgery has its place on the top round of the medical ladder.

11. The manufacturer makes out of certain materials a new product and offers it on the market for general and indiscriminate use, as for instance a brickmaker does.

12. Dentists are not, in the ordinary acceptance of the term, either mechanics or manufacturers, any more than the general surgeon. He applies his manufacture, such as the improvised splint or the pessary, as the case may be, cltering his product as the necessity of the case may demand, and does this through his professional knowledge of Anatomy, etc., aided by manipulative ability and mechanical skill. The dentist does precisely the same thing. The oculist adjusts and fits artificial eyes by fitting and grinding as the dentist does artificial teeth.

13. Artificial sets of teeth cannot be offered for sale giving each customer an opportunity to select what he likes; for no set of teeth could fit, or ever be fitted, to any but the one individual for whom it was especially made; therefore, no one can select a set of teeth as he might a hatchet or hand saw or any other manufactured articles in market.

14. Dental manufacturers are never classed by business men with dentists; we are a separate and distinct body of men.

15. The Census Report of 1880 declares, "That persons following individual pursuits shall be classed together," and we find in that Report headed, "persons engaged in selected occupations," Lawyers, Physicians, Dentists, Druggists, Preachers, etc., classed together. That in the Census of 1880 the precedent is established; and as a matter of fact under instructions from the Superintendent of the Census, General Francis A. Walker, the reports which dentists made in the city of New York were returned to them, and no further attempt was made to classify them as manufacturers, and we respectfully refer you to the correspondence between Dr. Thomas E. Gunning, of New York, and Gen'l Walker at that time.

16. The druggist from manufactured ingredients makes a new product to relieve suffering humanity. The dentist does the same thing.

17. We would call your attention to the fact, that all the materials used by the dentists, have or should have been classed under the head of "dental manufacturers." This is true of gold and other metals used in dental practice as well as of instruments, artificial teeth, rubber, etc.; there is no exception to this.

18. Many Dental Associations have forcibly expressed their sense of the great wrong done to the dental profession by being classed with manufacturers, when no other specialty of medicine is so treated.

We would appeal to your sense of fairness. We claim to be a respectable and law abiding profession and ask justice at your hands. For more than fifty-one years we have worked steadily and faithfully to gain our position and standing. This effort dates from the founding of the first College of Dental Surgery in the city of Baltimore.

We trust you will see the injustice of deposing us from our honorably acquired position as professional men, and relegating us to our starting point. We appeal to you, as officers of this great country to save us from this great wrong, which will undo the work of half a century.

You can readily replace us to where we were in the Census of 1880,in the list of "persons engaged in selected occupations." At this time all Dental Colleges in the United States are raising the standard of culture, and have increased the time required for preparation for graduation from two to three years.

A rigid examination has to be passed in Anatomy, Physiology, Pathology, Histology, Therapeutics, Materia Medica, Chemistry and Oral Surgery, before the Degree of Doctor of Dental Surgery is conferred. Is any such requirement made of any manufacturer ?

Leaving this statement in your hands, we feel confident, that when fully informed of the facts of the case, you will in your wisdom do justice to our profession.

Very respectfully,

ED. NELSON, D. D. S.,
R. B. WINDER, M.D., D.D.S.,
T. S. WATERS, D.D.S.,
A. J. VOLCK, PH.D., D.D.S.,
WM. A. MILLS, D.D.S.,
DAVID GENESE, D.D.S..
W. S. TWILLEY, D.D.S.,
*Committee.*

